# How relationship-maintenance strategies influence athlete burnout: Mediating roles of coach–athlete relationship and basic psychological needs satisfaction

**DOI:** 10.3389/fpsyg.2022.1104143

**Published:** 2023-01-09

**Authors:** Fenghui Fan, Jinyu Chen, Yunting Chen, Bing Li, Liya Guo, Yang Shi, Feng Yang, Qinjun Yang, Longfei Yang, Cody Ding, Huiying Shi

**Affiliations:** ^1^Faculty of Psychology, Southwest University, Chongqing, China; ^2^Virtual Laboratory of Sports and Health, Southwest University, Chongqing, China; ^3^Sports Psychology and Education Research Center, Southwest University, Chongqing, China; ^4^Chongqing Sports Technology Institute, Chongqing, China; ^5^Student Psychological Counseling Center, Chongqing Sports Technology Institute, Chongqing, China; ^6^Education Sciences and Professional Programs, University of Missouri–St. Louis, St. Louis, MO, United States

**Keywords:** athlete burnout, mental fatigue, relationship-maintenance strategies, coach–athlete relationship, basic psychological needs satisfaction

## Abstract

**Introduction:**

Athlete burnout has many potential negative effects on athletes’ sporting performance and careers. Maintaining and promoting the coach–athlete relationship to meet athletes’ basic psychological needs is one way to reduce burnout. Existing studies of the correlation between coach-athlete relationships and athlete burnout have mainly focused on the coaches’ leadership style, with little attention given to relationship-maintenance strategies and the mechanism of athlete burnout from the athletes’ perspective.

**Methods:**

Using an online survey of 256 adolescent athletes, we explore the relationship between relationship-maintenance strategies and athlete burnout, including the potential mediating effects of the coach–athlete relationship and basic psychological needs satisfaction.

**Results:**

(1) Athletes’ relationship-maintenance strategies negatively predicted athlete burnout. (2) Besides the direct effect, we found evidence to support three mediation paths: (a) the coach–athlete relationship, (b) basic psychological needs satisfaction, and (c) both as serial mediators.

**Discussion:**

These findings enhance understanding of the mechanism of athlete burnout, demonstrating the influence of factors beyond the coach’s role. The study also provides a theoretical basis for practical intervention by coaches, athletes, and sports organizations to reduce athlete burnout by focusing on athletes’ perspectives.

## 1. Introduction

The concept of mental fatigue originated in Freudenberger’s (1974) study of workers’ responses to stress in mental health care. Maslach and Jackson (1984) proposed the concept of burnout, describing the consumption of psychological resources caused by high-pressure work environments. A burnout model includes three dimensions (emotional exhaustion, depersonalization, and low scores on personal accomplishment; Maslach and Jackson, 1981). Although this model has been widely used in the study of job burnout, the concept is limited to the “people-work”; that is, the definition of general exhaustion is more applicable to the professional staff in human service with “provider—receiver” as the core characteristic. In sports, athletes focus on sports performance rather than a specific object. Therefore, Raedeke (1997) proposed the concept of athlete burnout. Athlete burnout has been described as a syndrome with three key dimensions: (a) emotional/physical exhaustion, a negative response to the intense demands of sports training and competition; (b) sport devaluation, the loss of interest and desire to participate in sports; and (c) reduced athletic accomplishment, a lower sense of achievement in one’s motor skills and abilities (Raedeke, 1997). Studies have shown that athlete burnout has increased over the past two decades (Madigan et al., 2022). Athlete burnout has many potential negative effects (Goodger et al., 2007; Gustafsson et al., 2017; Eklund and DeFreese, 2020), including lack of motivation, decreased engagement, poor sports performance, deterioration of personal relationships, and increased risk of depression (Cresswell and Eklund, 2005; Jowett et al., 2016; Smith et al., 2019). Athletes who suffer from these effects may consequently avoid training and competition or even leave their sport forever (Hu and Xu, 2008; Larson et al., 2019). Therefore, the influencing factors of athlete burnout need to be investigated to understand the mechanism of athlete burnout better. By expanding the theoretical perspective on what causes athlete burnout, practical ideas can be offered for reducing athlete burnout in athletes.

Athlete burnout is affected by both endogenous and exogenous factors (Zhang et al., 2006). Studies have shown that the maladaptive profile of perfectionism (Yook and Shin, 2014; Olsson et al., 2022), low level of openness (Li et al., 2018; Garcia-Hernandez et al., 2020), low mental toughness (Wang et al., 2014; Guo et al., 2021), and other endogenous factors have significant positive correlations with athlete burnout. Exogenous factors found to significantly predict burnout include the pressure of sports competition (Yang, 2018; Lin et al., 2021), the pressure within sports organizations (Wu et al., 2021; Wu, 2022), and lack of social support (Lu et al., 2016; Martinez-Alvarado et al., 2021; Shang and Yang, 2021). In the context of China’s competitive sports, athletes participate in intense training throughout the year and spend much time outside of training with teammates and coaches. Compared with individual factors, external factors such as social and organizational factors may be more important factors in predicting athlete burnout (Zhang, 2010). This view coincides with the Self-Determination Theory (SDT) proposed by Deci and Ryan (1985). According to SDT, individuals tend to grow and perfect their own personality and have three basic psychological needs for self-integration (autonomy, competence, and relatedness). The satisfaction of these three basic psychological needs is subject to external environment. When the environmental factors satisfy psychological needs, individuals will develop positively and healthily (Deci and Ryan, 2017). Athletes will make free choices of actions based on awareness of their own needs and social environment (Deci and Ryan, 2000). The proposal of SDT provides a new theoretical framework for athlete burnout. Research on athlete burnout has shifted attention from the individual to social factors (Sun and Zhang, 2012; Davis et al., 2019b). Therefore, based on SDT, we explore the social factors potentially affecting athlete burnout (Lonsdale et al., 2009).

Studies have shown that the coach–athlete relationship is an important social predictor of athlete burnout (Cresswell and Eklund, 2007; Isoard-Gautheur et al., 2016). In the social life of athletes, the relationship with their coach is an important interpersonal structure (Xie, 2018), entailing two-way interactions. However, most studies of the correlation between the coach–athlete relationship and athlete burnout have only explored from the perspective of coaches’ leadership style (Gustafsson et al., 2008; Barcza-Renner et al., 2016; Cho et al., 2019).

From the perspective of athletes, maintaining and promoting the coach–athlete relationship is an important part of sports training. If athletes can interact effectively with coaches and form high-quality relationships through relationship-maintenance strategies, they will more likely feel that their psychological needs are being met, thus reducing the risk of athlete burnout (Choi and JungKooin, 2020). Therefore, focusing on athletes’ perspective, this study adopts SDT as the theoretical framework to explore athletes’ relationship-maintenance strategies, the coach–athlete relationship, essential psychological needs satisfaction, and athlete burnout.

## 2. Literature review and hypothesis development

### 2.1. Relationship-maintenance strategies and athlete burnout

Relationship-maintenance strategies are the behaviors adopted by peers to maintain satisfactory relationship quality, for instance, in terms of commitment or love (Stafford and Canary, 1991). For coaches and athletes, relationship-maintenance strategies refer to respective efforts to maintain emotional closeness, cognitive commitment, and behavioral complementarity (Guo, 2016). Unless both parties use effective relationship-maintenance strategies, their connection will be weak (Canary and Stafford, 1994), and the additional time and energy athletes invest in maintaining the relationship will lead to burnout. SDT believes that the positive interaction between individuals and the social environment contributes to satisfying basic psychological needs, thus alleviating their negative feelings (Liu Y. et al., 2022). The relationship maintenance strategies athletes adopt to repair, stabilize or improve their relationship with coaches is a kind of positive interaction. The good coach–athlete relationship brought by such positive interaction is an important part of the harmonious social environment. When athletes’ basic psychological needs are met in the environment, their happiness will be improved (Milyavskaya and Koestner, 2011; Ye et al., 2019), and athlete burnout will be reduced (Rhind and Jowett, 2010a).

Based on the seven types of relationship-maintenance strategies proposed by Rhind and Jowett (2010a,b) and Guo (2016) proposed six dimensions of relationship-maintenance strategies for Chinese athletes: conflict management, communication, motivation, support, assurance, and social networks. Conflict management refers to identifying, discussing, resolving, and monitoring conflicts in the relationship. Conflict management style significantly impacts athlete burnout: compromise, cooperation, and compliance have been shown to alleviate burnout, whereas the opposite effect was found for avoidance (Eun-Young, 2015). Coaches’ management of conflicts through communication can also help to enhance athletes’ willingness to continue participating in sports (Kim and Cho, 2022). The communication dimension refers to athletes and coaches sharing information and feedback on any problems in training and their personal life. This communication should be two-way; a democratic coach will consider athletes’ views and make timely adjustments to the training program. Such democratic behavior by the coach can produce a positive emotional experience for athletes, further enhancing their engagement and reducing athlete burnout (Xie and Yao, 2010; Gao et al., 2021). Open communication is crucial in promoting the relationship between coaches and athletes. In studies of Spanish athletes (Garcia-Hernandez et al., 2020) and Chinese athletes (Li et al., 2018), a low level of openness was found to be characteristic of athletes with a high degree of burnout. Motivation refers to the intention of coaches and athletes to persist in pursuing goals. Interpersonal relationships in sports are usually intention- and results-oriented (Choi et al., 2020), and motivation has been found to predict athlete burnout negatively (Graña et al., 2021; Madigan et al., 2022). Support refers to helping others through difficulties in sports and life. Research shows that coaches’ autonomy support can reduce athlete burnout by enhancing intrinsic motivation (He et al., 2021; Kang et al., 2021). Supportive coaching styles are especially effective for athletes with low self-esteem (Conroy and Coatsworth, 2006). Assurance means that the coach and athlete trust each other, believe they can perform well in sports training and personal life, and convey to the other that they will continue the relationship. Assurance indicates the athlete’s emotional sustenance to their coaches and affirmation of the coach’s ability, which enables athletes and coaches to reach a consensus on the team goal, and improve team cohesion (Yang et al., 2014). High-level team cohesion is one of the key factors for team success (Liang and Li, 2016), which negatively predicts athlete burnout (Chang et al., 2019; Pacewicz et al., 2020). Social network refers to the communication between coaches and athletes in training and life. A positive social network in the social environment of sports can reduce athlete burnout (Pacewicz et al., 2019). On the contrary, negative social interactions, such as rejection or neglect, will lead to athlete burnout (DeFreese and Smith, 2014).

Drawing on SDT, we first hypothesized that relationship-maintenance strategies negatively predict athlete burnout.

### 2.2. The mediating roles of the coach–athlete relationship and basic psychological needs satisfaction

As one of the most important interpersonal relationships for athletes, the coach–athlete relationship notably influences athletes’ performance and even athlete burnout (Guo et al., 2021). Within this unique interpersonal relationship, the feelings, thoughts, and behaviors of coaches and athletes are mutually and causally interconnected (Jowett and Meek, 2000; Jowett and Ntoumanis, 2004; Adie and Jowett, 2010). This definition emphasizes the bidirectional and dynamic nature of the coach–athlete relationship, highlighting both sides’ interdependence and mutual influence. These feelings, thoughts, and behaviors have been reflected in Jowett’s (2007) 3Cs model, which holds that the relationship between coaches and athletes includes closeness, commitment, and complementarity. Closeness reflects the affective meanings the coach and athlete assign to their relationship (e.g., respect, trust, liking). Commitment is the intention to maintain a long-term partnership within the focal sport. Finally, complementarity refers to the complementary or cooperative interactions between coach and athlete during training. Research shows that the quality of the coach–athlete relationship is a typical organizational stressor in the context of athletes’ training and competition. When this relationship is harmonious, athletes not only avoid interpersonal pressure but also benefit from coaches’ help and support, thus reducing the likelihood of experiencing burnout symptoms (Tabei et al., 2012). Conversely, a lower-quality coach–athlete relationship will increase athlete burnout (Isoard-Gautheur et al., 2016; Ruser et al., 2021; JungKooin et al., 2022; Kim and Cho, 2022). Therefore, improving the quality of this relationship should reduce the risk of athletes experiencing burnout. Jowett and Poczwardowski (2007) proposed that the quality of the coach–athlete relationship is determined by the interpersonal communication between coaches and athletes, with two-way communication positively impacting the relationship (Choi et al., 2020). This communication depends somewhat on relationship-maintenance strategies, which have been found to partly explain differences in the closeness, commitment, and complementarity dimensions of the coach–athlete relationship. Openness and social networks are positively correlated with closeness; motivation and support are positively related to commitment; and assurance is positively related to complementarity (Rhind and Jowett, 2011). Beyond exercise, intimacy research has shown that effective relationship-maintenance strategies facilitate stable, long-term, and satisfying intimate relationships (Canary and Stafford, 1994; Ogolsky and Bowers, 2013). Thus, we predict that high-quality relationship-maintenance strategies can maintain effective coach–athlete relationships, reducing athlete burnout.

*H2*: The coach–athlete relationship mediates the relationship between athletes’ relationship-maintenance strategies and athlete burnout.

Self-Determination Theory suggests that basic psychological needs satisfaction is an important concept in explaining healthy participation in sports (Ryan and Deci, 2000). Meeting the three basic needs of autonomy, competence, and relatedness is necessary for individuals to achieve their potential, flourish, and avoid unhealthy or maladaptive states (e.g., athlete burnout; Sheldon and Niemiec, 2006). For athletes, autonomy is satisfied by being able to act on their own will and values when training and competing; competence needs are met by improving performance, meeting higher challenges, and having opportunities to develop their sports skills; and relatedness needs are satisfied through social support networks and strong interpersonal connections within the sporting community (Vallerand and Rousseau, 2001). Research suggests that basic psychological needs satisfaction promotes autonomous motivation, positive emotions, well-being, and athletic performance in athletes, whereas non-satisfaction is associated with physical health disruption, lack of motivation, and decreased well-being (Tay and Diener, 2011; Ryan and Deci, 2017; Lopes and Vallerand, 2020). Quested and Duda (2010) highlighted personal qualities that may facilitate or hinder basic psychological needs satisfaction as the main predictors of maladaptive outcomes such as athlete burnout. Satisfying their basic psychological needs helps protect athletes from high levels of burnout symptoms (Lonsdale et al., 2009; Quested and Duda, 2011; Li et al., 2013; Jowett et al., 2016). Basic psychological needs satisfaction is influenced by situational factors such as social support and organizational environment (Van den Broeck et al., 2016; Coxen et al., 2021). The positive social environment created by a good coach–athlete relationship helps to satisfy athletes’ basic psychological needs, thereby supporting their long-term sport participation and well-being (Chu and Zhang, 2019) and reducing athlete burnout. We propose that athletes can obtain social support and improve relationship satisfaction by using relationship-maintenance strategies in interactions with their coaches, thus satisfying their basic psychological needs and, in turn, reducing burnout (Rocchi and Pelletier, 2018; Nascimento et al., 2019).

*H3*: Basic psychological needs satisfaction mediates the relationship between athletes’ relationship-maintenance strategies and athlete burnout.

According to SDT, athlete burnout result in basic psychological needs not being satisfied, which hinders the internalization of external motivation, and leads to the lack of motivation, enthusiasm, and interest of athletes (Sun and Zhang, 2012). Therefore, satisfying basic psychological needs is a prerequisite for preventing and alleviating athlete burnout, while the social environment is an external determinant of basic psychological needs satisfaction (Wu et al., 2018). The social environment usually involves people close to the individual, such as parents, teachers, and coaches (Deci and Ryan, 2012). For athletes, bonding with their coach is an important social relationship in training and competition (Ai and Wang, 2017). In addition, the 3Cs model theorizes that the coach–athlete relationship is a medium for satisfying both parties’ basic psychological needs (Jowett, 2005, 2007). An athlete’s perception of the quality of the coach–athlete relationship is an important determinant of basic psychological needs for satisfaction (Riley and Smith, 2011; Choi et al., 2013; Felton and Jowett, 2013b), self-determined motivation (Riley and Smith, 2011), and well-being (Felton and Jowett, 2013b). Felton and Jowett (2013a,b, 2015) conducted a series of studies demonstrating the predictive utility of social relationships in sports (i.e., secure coach attachment, perceived quality of the coach–athlete relationship) for basic psychological needs satisfaction and subsequent well-being. As relationship quality cannot be maintained without effective relationship-maintenance strategies (Canary and Stafford, 1994; Rhind and Jowett, 2010b), we predict that the coach–athlete relationship and basic psychological needs satisfaction are serial mediators.

**Figure 1:**
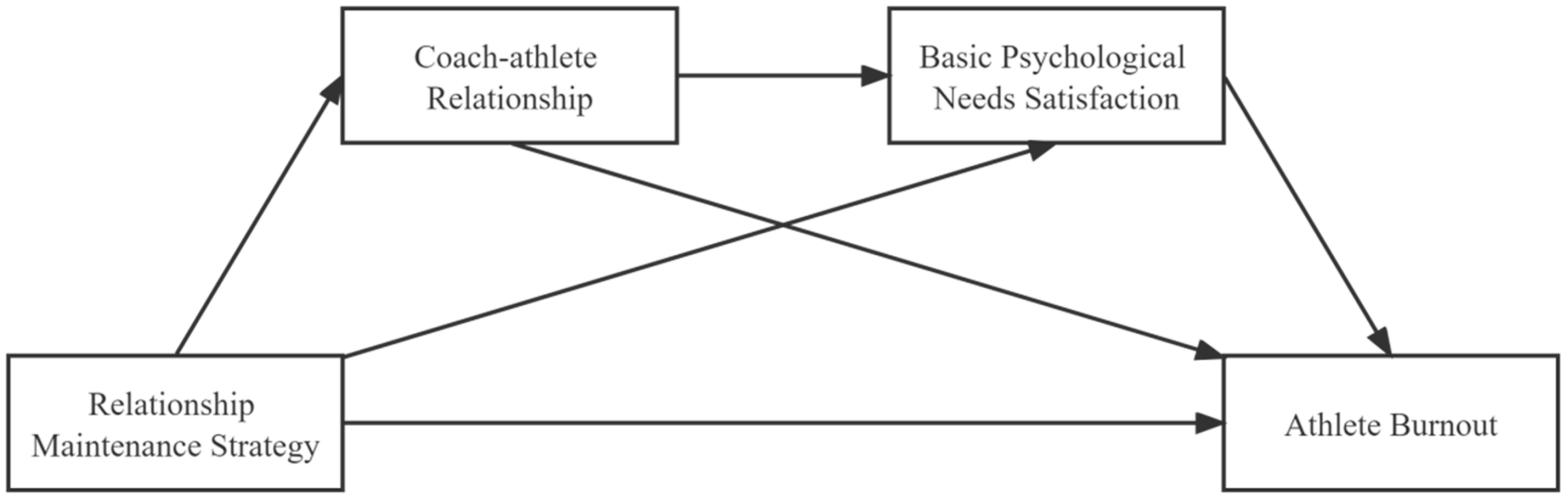
Proposed sequential mediation model.

*H4*: The coach–athlete relationship and basic psychological needs satisfaction serially mediate between athletes’ relationship-maintenance strategies and athlete burnout. Our research model is outlined in Figure 1.

## 3. Materials and methods

### 3.1. Participants

Convenience sampling was used to select 272 athletes from various sports teams in Chongqing, China, to complete the online questionnaire survey. Sixteen participants were excluded from the data analysis due to inconclusive responses, too many missing values, and short response durations (Gröschke et al., 2022). The valid response rate was 94.1%. Of the 256 participants whose responses were analyzed, 171 (66.8%) were male, and 85 (33.2%) were female. In terms of athletic prowess, 87 (34.0%) were national first-class athletes or above, 76 (29.7%) were national second-class athletes, and 93 (36.3%) were below that level. The average age was 19.95 ± 3.22 years, while the average number of training years was 3.17 ± 2.91 years. Participants’ focal sports included race walking, taekwondo, boxing, and tennis.

Harman’s single-factor test was used to assess possible common method bias. The results showed that 14 factors had characteristic roots greater than 1, and the unrotated first factor explained only 33.9% of the total variation. As this value is below the threshold of 40%, there appears to be no significant common method bias in this study (Podsakoff et al., 2003).

### 3.2. Methods

#### 3.2.1. The coach–athlete relationship maintenance questionnaire (CARM-Q)

Developed by Guo (2016), the CARM-Q comprises 29 items across six subfactors: communication (e.g., “I am willing to share my emotions with my coach”), motivation (e.g., “I work out challenging tasks”), support (e.g., “I help the coach when he/she is in trouble”), conflict management (e.g., “I try to keep myself in check when I disagree”), social networks (e.g., “we have a lot of mutual friends”), and assurance (“I let the coach know he/she can count on me”). Participants respond to each item on a seven-point Likert scale, ranging from 1 (“completely inconsistent”) to 7 (“completely consistent”). Higher scores indicate that the athlete engages more extensively in relationship-maintenance strategies with respect to their coach. Cronbach’s α was 0.96 for the total questionnaire and 0.79, 0.88, 0.85, 0.79, 0.82, and 0.69 for each respective subfactor.

#### 3.2.2. The coach–athlete relationship questionnaire (CART-Q)

Developed by Jowett and Ntoumanis (2004), the CART-Q includes 11 items across three subfactors: closeness (e.g., “I like my coach”), commitment (e.g., “I feel loyal to my coach and am willing to maintain a long-term cooperative relationship with him/her”), and complementarity (e.g., “When I am coached by my coach, I adopt a friendly stance”). Again, participants responded to each item on a seven-point Likert scale, ranging from 1 (“completely inconsistent”) to 7 (“completely consistent”). Higher scores indicate a higher quality coach–athlete relationship. Cronbach’s α was 0.94 for the total questionnaire and 0.84, 0.86, and 0.88 for each respective subfactors.

#### 3.2.3. The basic needs satisfaction In sport scale (BNSSS)

The BNSSS was developed by Ng et al. (2011) and includes 20 items across three subscales: autonomy needs (e.g., “I feel I am doing what I really want to do”), competence needs (e.g., “I have proficient skills in my sport”), and relatedness needs (e.g., “I care about others”). Once again, responses to each item were given on a seven-point Likert scale, ranging from 1 (“completely inconsistent”) to 7 (“completely consistent”). Higher scores indicate greater satisfaction of basic psychological needs in the given dimension (with item 14 reverse-scored). Cronbach’s α was 0.93 for the total questionnaire and 0.91, 0.89, and 0.91 for each subscale.

#### 3.2.4. Athlete burnout questionnaire (ABQ)

Developed by Raedeke and Smith (2001), the ABQ comprises 15 items across three subfactors: emotional/physical exhaustion (e.g., “I feel overly tired from my sport participation”), reduced athletic accomplishment (e.g., “It seems that no matter what I do, I cannot do my best”), and sport devaluation (e.g., “The energy I expend on training for a game might be better used doing something else”). Participants responded to each item on a five-point Likert scale, ranging from 1 (“never”) to 5 (“always”). Higher scores indicate a higher degree of athlete burnout (with items 1 and 14 reverse-scored). Cronbach’s α was 0.83 for the total questionnaire and 0.85, 0.52, and 0.74 for each subfactor.

### 3.3. Statistical analysis

Descriptive statistics analysis and correlation analysis of the data were performed in SPSS 25.0. Hierarchical regression analysis was carried out to investigate predictive relationships between variables. We used Hayes (2013) SPSS macro PROCESS (Model 6) to calculate the confidence intervals and effect values of the direct effects between relationship maintenance strategies and athlete burnout, as well as the indirect effects of relationship maintenance strategies on athlete burnout through the coach–athlete relationship and basic psychological needs satisfaction. The bootstrapping method with robust standard errors was employed to test the significance of the effects (Hayes, 2013). The bootstrapping method produced 95% bias-corrected confidence intervals (CIs) of these effects from 5,000 resamples of the data. If CIs did not include zero, the effects in Model 6 were significant at *α* = 0.05. All statistical tests were two-tailed.

## 4. Results

### 4.1. Descriptive statistics and correlation analysis of variables

We analyzed the correlations between athletes’ relationship-maintenance strategies, coach–athlete relationship, basic psychological needs satisfaction, and athlete burnout.

As reported in Table 1, the results show that relationship-maintenance strategies were significantly negatively correlated with athlete burnout (*r* = −0.52, *p* < 0.01) but significantly positively correlated with the coach–athlete relationship (*r* = 0.71, *p* < 0.01) and basic psychological needs satisfaction (*r* = 0.70, *p* < 0.01). The coach–athlete relationship and basic psychological needs satisfaction were both significantly negatively correlated with athlete burnout (*r* = −0.47, *p* < 0.01; *r* = −0.57, *p* < 0.01). The data meet the statistical requirements for further analysis of the mediating effects of the coach–athlete relationship and basic psychological needs satisfaction (Wen and Ye, 2014).

**Table 1:** Descriptive statistics and correlation analysis.

Variables	M ± SD	1	2	3	4
1. CARM	5.22 ± 0.96	1			
2. CAR	6.14 ± 0.92	0.71^**^	1		
3. BNS	2.38 ± 0.54	0.70^**^	0.56^**^	1	
4. AB	5.28 ± 0.93	−0.52^**^	−0.47^**^	−0.57^**^	1

CARM, coach–athlete relationship maintenance; CAR, coach–athlete relationship; BNS, basic psychological needs satisfaction; AB, athlete burnout. M ± SD, mean ± standard deviation. ^**^p < 0.01.

### 4.2. Regression analysis

We next analyzed the predictive relationships between relationship-maintenance strategies, coach–athlete relationship, basic psychological needs satisfaction, and athlete burnout.

As presented in Table 2, the regression results show that relationship-maintenance strategies negatively predicted athlete burnout (*β* = −0.47, *p* < 0.001), supporting Hypothesis 1. Relationship-maintenance strategies positively predicted the coach–athlete relationship (*β* = 0.71, *p <* 0.001) and basic psychological needs satisfaction (*β* = 0.61, *p* < 0.001), while the coach–athlete relationship positively predicted basic psychological needs satisfaction (*β* = 0.13, *p <* 0.05) and negatively predicted athlete burnout (*β* = −0.15, *p* < 0.05). Finally, basic psychological needs satisfaction negatively predicted athlete burnout (*β* = −0.38, *p* < 0.001).

**Table 2:** Regression analysis of variables in the model.

Regression		Model index			Coefficients	
Outcome variable	Independent variable	*R*	*R* ^2^	*F*	*β*	*t*
AB	CARM	0.47	0.22	72.58^***^	−0.47	−8.52^***^
CAR	CARM	0.71	0.50	252.42^***^	0.71	15.89^***^
BNS	CARM	0.71	0.50	128.56^***^	0.61	9.76^***^
	CAR				0.13	2.12^*^
AB	CARM	0.61	0.36	48.63^***^	−0.14	−1.72
	CAR				−0.15	−2.16^*^
	BNS				−0.38	−5.39^***^

CARM, coach–athlete relationship maintenance; CAR, coach–athlete relationship; BNS, basic psychological needs satisfaction; AB, athlete burnout. ^*^p < 0.05; ^***^p < 0.001; *N* = 256; *β* = standardized coefficients.

### 4.3. Mediating effect test

The Bootstrap method was adopted in this study to analyze the mediating effect, with relationship maintenance strategy as the independent variable, coach–athlete relationship and basic psychological needs satisfaction as the mediating variable, and athlete burnout as the dependent variable.

Table 3 reports the results of the mediating effect test. The coach–athlete relationship partially mediated between relationship-maintenance strategies and athlete burnout (95% CI = [−0.21, −0.02]); the effect value is −0.11, accounting for 21% of the total effect. Hypothesis H2 is thus supported. Basic psychological needs satisfaction is also partially mediated between relationship-maintenance strategies and athlete burnout (95% CI = [−0.43, −0.09]); the effect value is −0.23, accounting for 44% of the total effect. Therefore, Hypothesis 3 is also supported.

**Table 3:** Mediating effect test.

Effect type	Path	Effect	Proportion of total effect	Bootstrap 95% CI	
				LLCI	ULCI
Indirect	CARM→CAR→AB	−0.11	21%	−0.203	−0.015
	CARM→BNS → AB	−0.23	44%	−0.430	−0.093
	CARM→CAR→BNS → AB	−0.04	7%	−0.080	−0.005
Direct effect	CARM→AB	−0.14	27%	−0.306	0.021
Total effect		−0.52		−0.627	−0.417

CARM, coach–athlete relationship maintenance; CAR, coach–athlete relationship; BNS, basic psychological needs satisfaction; AB, athlete burnout. Bootstrap sample size = 5,000. CI, confidence interval; LLCI, lower limits of confidence interval; ULCI, upper limits of confidence interval.

The coach–athlete relationship and basic psychological needs satisfaction play a serial mediating role between relationship-maintenance strategies and athlete burnout (95% CI = [−0.08, −0.00]); the effect value is −0.04, accounting for 7% of the total effect. The results supported Hypothesis 4.

**Figure 2:**
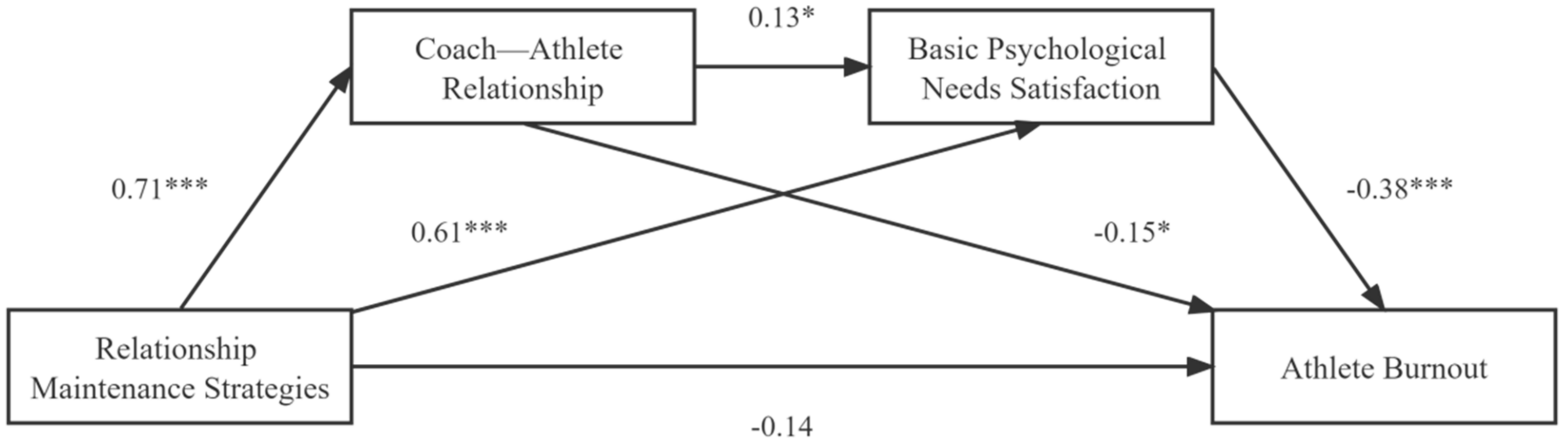
Associations between relationship-maintenance strategies and athlete burnout for the whole sample (*N* = 256). *p < 0.05, ***p < 0.001.

The research model, including effect values, is shown in Figure 2.

## 5. Discussion

Drawing on SDT, this study explores the mechanism of the association between relationship-maintenance strategies and athlete burnout. The results show the negative predictive effect of relationship-maintenance strategies on athlete burnout, as well as the individual and serial mediating effects of the coach–athlete relationship and basic psychological needs satisfaction. The study thus reveals three mediation paths, indicating that athletes who use more relationship-maintenance strategies will perceive a higher quality coach–athlete relationship and meet more of their basic psychological needs, thereby reducing the risk of athlete burnout.

### 5.1. The negative predictive effect of relationship-maintenance strategies on athlete burnout

The results show that athletes’ relationship-maintenance strategies negatively predict athlete burnout. The relationship maintenance behavior of athletes can create a positive social interaction environment in which athletes are less likely to experience burnout during sports because their basic psychological needs are met. Regarding the specific dimensions of these strategies, conflict management and motivation strategies reflect athletes’ efforts to clarify their own and the coaches’ expectations and express their intention to continue cooperating with coaches (Rhind and Jowett, 2010b). These strategies can reduce the incidence and severity of conflicts between athletes and coaches and help athletes maintain full enthusiasm for their sport to avoid excessive emotional exhaustion and negative evaluation of sports. The assurance strategy reflects athletes taking a positive approach to problems in training and life, ensuring coaches can trust them to deal with them well (Guo, 2016). This strategy dimension also focuses on preserving athletes’ high evaluation of their own abilities, which helps them avoid losing a sense of accomplishment. Communication, support, and social network strategies all reflect the importance to athletes of establishing supportive relationships and maintaining effective communication with their coaches regarding sports and non-sports matters (Rhind and Jowett, 2010b). These strategies also help athletes relieve their negative emotions regarding sports. Therefore, whereas previous studies emphasized the coach’s responsibility for creating a good atmosphere and thereby minimizing athlete burnout, we demonstrate the importance of athletes’ own relationship-maintenance strategies. In particular, it is essential for athletes to actively and openly communicate with their coaches about personal troubles in training and life and to obtain coaches’ help and advice.

### 5.2. The mediating roles of coach–athlete relationship and basic psychological needs satisfaction

The study’s results show that athletes’ relationship-maintenance strategies indirectly affect athlete burnout through coach–athlete relationship and basic psychological needs satisfaction, as individual and serial mediators. These findings indicate that the athlete’s relationship with their coach and satisfaction of basic psychological needs are important ways for relationship-maintenance strategies to reduce the athlete’ s risk of burnout. These insights are consistent with the results of previous studies (Isoard-Gautheur et al., 2016; Kent et al., 2018; Ariani, 2019; Davis et al., 2019a).

Athletes who use appropriate relationship-maintenance strategies will show respectful and friendly attitudes during interactions with their coaches, thereby contributing to the development and maintenance of good interpersonal relationships (LaVoi, 2007) and enhancing the quality of the coach–athlete relationship (Davis et al., 2019a; Choi et al., 2020). When athletes feel close to coaches and appreciate one another (closeness), believe they can work with their coaches (complementarity), and intend to maintain a long-term partnership (commitment), they will be less likely to experience feelings of reduced athletic accomplishment, emotional exhaustion, and sport devaluation (Cresswell and Eklund, 2007; Gustafsson et al., 2008; Isoard-Gautheur et al., 2016). Thus, athletes’ relationship-maintenance strategies can predict athlete burnout through the mediating role of the coach–athlete relationship.

Athletes who use relationship-maintenance strategies are more proactive in their interactions with coaches, facilitating the satisfaction of three basic psychological needs: autonomy, competence, and relatedness (Mo and Pi, 2020). When their basic psychological needs are satisfied, athletes are less inclined to devalue their sport and have a greater sense of belonging to the sporting environment (Ariani, 2019). They also strongly believe they can achieve good results and rationally allocate training time to avoid excessive physical consumption (Kent et al., 2018). The basic psychological needs satisfaction causes athletes to experience less sports devaluation, reduced athletic accomplishment, and emotional/physical exhaustion, and the level of burnout decreases accordingly (Kent et al., 2018; Liu M. et al., 2022; Shannon et al., 2022). Thus, athletes’ relationship-maintenance strategies can predict athlete burnout by mediating basic psychological needs satisfaction.

Based on the finding that the coach–athlete relationship and basic psychological needs satisfaction function as serial mediators, the study provides new evidence that athletes’ psychological needs are satisfied through the coach–athlete relationship, consistent with Jowett and Timson-Katchis (2005; see also Jowett and Shanmugam, 2016). Athletes establish and sustain good relationships with coaches through relationship-maintenance strategies. When the coach–athlete relationship is harmonious, athletes may gain more respect from coaches, satisfying their relatedness needs. With more support and help from coaches, athletes will also perceive a greater sense of control, satisfying their autonomy needs. Through their efforts to promote mutual trust with coaches, athletes will participate more actively in training and competition, facilitating continuous improvement in their abilities and, thus, satisfying their competence needs (Riley and Smith, 2011; Choi et al., 2013). In addition, SDT considers motivation as a continuum of self-determination and holds that basic psychological needs satisfaction contributes to the internalization of external motivation, and the degree of internalization is determined by the degree of satisfaction of psychological needs (Ryan and Deci, 2000). Therefore, basic psychological needs satisfaction will increase the level of athletes’ self-determination motivation to participate in training or competition and avoid externalization and loss of athletic motivation (Adie and Jowett, 2010). As athletes maintain a positive psychological state (Ye et al., 2016), burnout is prevented or alleviated.

Three new ideas emerged from this study. First, we provide a new perspective on athlete burnout by revealing the positive associations among athletes’ relationship-maintenance strategies, the coach–athlete relationship, and the satisfaction of basic psychological needs. Second, this study found that the antecedent role of the coach–athlete relationship in basic psychological needs satisfaction, that is, the positive influences of coach–athlete relationship on basic psychological needs satisfaction, suggesting that the direction of this relationship should be taken into account. Third, whereas most previous studies have examined how coaches’ behaviors influence athlete burnout, this study highlights the importance of athletes’ own relationship-maintenance behaviors in preventing burnout. Compared to previous findings before the COVID-19 epidemic (Isoard-Gautheur et al., 2016; McGee and Defreese, 2019; Davis et al., 2019a), our study demonstrated that athletes’ relationship maintenance strategies still predicted coach–athlete relationship quality, and athletes who perceived strong coach–athlete relationship experienced less burnout, suggesting the importance of athletes’ relationship maintenance strategies in preventing athlete burnout in different social contexts.

Our findings have important practical implications for promoting athlete burnout interventions from athletes, coaches, and sports organizational management. First, athletes’ training in sports skills should be combined with psychological training focused on improving interpersonal relationships. Through learning how to use appropriate relationship-maintenance strategies, athletes will become better equipped to cultivate high-quality interpersonal relationships, helping satisfy their basic psychological needs and creating a more comfortable environment for their sporting activities. Second, coaches should observe and improve interaction patterns with athletes and seek to maintain a good coach–athlete relationship. For athletes with unmet autonomy needs, coaches should give them a certain degree of freedom to decide on training activities in preparation for competitions. For athletes with unmet competence needs, coaches should help them create successful experiences and enhance their self-efficacy. For athletes with unmet relatedness, coaches should ensure they receive more education on interpersonal skills. Third, sports organizational management should intervene in difficult relationships between athletes and coaches to find solutions and prevent athlete burnout. Furthermore, in order to eliminate the negative effects of athlete burnout, policy support and guidelines should be adequately provided so that athletes and coaches, as well as management, are aware of the importance of maintaining good coach–athlete relationship and actively engage in relevant training.

### 5.3. Limitations and future research avenues

This study has several limitations. First, the sample size hinders the research, as the small number of Chinese athletes recruited in Chongqing could hardly represent all athletes in China. Future studies should contain more convincing samples, especially athletes in different sports, to better support the present findings and possibly provide further insight into the study on athlete burnout. Second, this study is a cross-sectional investigation, which made it impossible for us to deduce the causal relationships between research variables. Therefore, a longitudinal study in the future needs to elucidate the causal relationship between relationship-maintenance strategies and athlete burnout. Third, because all the questionnaire responses were self-reported by athletes, the results may be affected by endogeneity bias. It would benefit future studies to include other measures, such as in-depth observations and coach-reported data. Fourth, this study does not consider the cultural universality of interpersonal relationships. In Chinese culture, coach–athlete relationship is characterized by a typical paternalistic leadership, a tendency of absolute worship and obedience of juniors to seniors and subordinates to superiors based on blood ties and ranks. This differs from the characteristic of coach–athlete relationships in Western culture, which emphasizes contractual elements. Cross-cultural research should be conducted to verify the role of coach–athlete relationship in athlete burnout.

## Data availability statement

The original contributions presented in the study are included in the article/supplementary material, further inquiries can be directed to the corresponding authors.

## Ethics statement

The studies involving human participants were reviewed and approved by the Ethics Committee at the Faculty of Psychology Southwest University. Written informed consent to participate in this study was provided by the participants’ legal guardian/next of kin.

## Author contributions

FF: conceptualization, methodology, investigation, and funding acquisition. JC: writing — original draft and investigation. YC: data curation, writing — review and editing. BL and LG: resources and validation. YS: resources and project administration. FY: validation. QY: data curation. LY: investigation. CD: conceptualization, supervision, and writing – review and editing. HS: project administration, methodology, writing – review and editing, and funding acquisition. All authors contributed to the article and approved the submitted version.

## Funding

This research was funded by the Major Project of Chongqing Sports Bureau (A202211, A202022, and A202119), the Chongqing Sports Scientific Research Project (B2019008), and National Social Science Foundation Research Program (Grant No. 21BSH117).

## Conflict of interest

The authors declare that the research was conducted in the absence of any commercial or financial relationships that could be construed as a potential conflict of interest.

## Publisher’s note

All claims expressed in this article are solely those of the authors and do not necessarily represent those of their affiliated organizations, or those of the publisher, the editors and the reviewers. Any product that may be evaluated in this article, or claim that may be made by its manufacturer, is not guaranteed or endorsed by the publisher.
